# Prospective association of a Lifestyle Risk Factor Index with type 2 diabetes in the Multiethnic Cohort

**DOI:** 10.1007/s00394-025-03721-x

**Published:** 2025-06-16

**Authors:** Simone Jacobs, Rebecca Klapp, Yurii B. Shvetsov, Bruce S. Kristal, Veronica Wendy Setiawan, Loïc Le Marchand, Gertraud Maskarinec

**Affiliations:** 1 University of Hawaii Cancer Center, Honolulu, HI USA; 2 University of Southern California, Los Angeles, CA USA; 3 Jean Mayer USDA Human Nutrition Research Center on Aging at Tufts University, Boston, MA USA; 4 Max Delbrück Centrum für Molekulare Medizin, Berlin, Germany; 5 IU International University of Applied Sciences, Erfurt, Germany

**Keywords:** Type 2 diabetes, Ethnicity and race, Longitudinal, Lifestyle, Cohort studies

## Abstract

**Purpose:**

This study examined behaviors captured in a composite Lifestyle Risk Factor Index (LSRI) in relation to type 2 diabetes (T2D) incidence across five ethnic groups in the Multiethnic Cohort, considering the cumulative and interactive effects of lifestyle factors.

**Methods:**

The study population included 165,383 European American (EA), African American (AA), Native Hawaiian (NH), Japanese American (JA), and Latino (L) participants. The LSRI score, assessed by baseline questionnaire, assigns 1 point each for no current smoking, physical activity (≥ 150 min/week), consuming < 1 (women) or < 2 (men) alcoholic drinks/day and adhering to ≥ 3 of 7 dietary recommendations. Hazard ratios with 95% confidence intervals were estimated by Cox regression.

**Results:**

During a mean follow-up of 17 years, 44,518 (27%) incident T2D cases were identified. Adherence was highest for moderate alcohol (86%) and no current smoking (84%), followed by physical activity (81%) and diet (22%). A 1-point increase in LSRI was associated with a 6% lower incidence of T2D (HR = 0.94; 95%CI 0.93–0.95) in the BMI-adjusted model. No current smoking, physical activity, and healthy diet (without BMI adjustment only) were inversely and moderate alcohol consumption positively associated with T2D incidence. The LSRI was associated with lower T2D risk in BMI-adjusted models for participants with AA, L, and EA ancestry and among JA before BMI adjustment.

**Conclusions:**

These results confirm that a combination of lifestyle behaviors is critical in T2D prevention. However, not all LSRI components impact T2D risk equally, and both, associations and the impact of BMI adjustment, vary by ethnic group.

## Introduction

The occurrence of type 2 diabetes mellitus (T2D) constitutes a significant global public health crisis due to its increasing prevalence and associated complications. The International Diabetes Federation (IDF) estimated that the prevalence of diabetes among those in the age group 20–79 years stood at 10.5% in 2021, affecting 536.6 million people worldwide. This number is projected to rise to 12.2% by 2045, equating to an estimated 783.2 million people [1]. Of these cases, over 90% involve T2D, indicating that more than 483 million people had T2D in 2021. As the most common form of diabetes, T2D has a substantial impact compared to other types. It is associated with various complications, such as chronic kidney disease, eye damage and peripheral neuropathy (causing amputation of lower extremity), as well as with significantly higher all-cause and cardiovascular disease mortality [2, 3]. Moreover, T2D has a significant economic impact, manifested through direct medical expenses as well as indirect costs (e.g., due to incapacity for work and shorter life expectancy) [4]. In light of this, urgent measures are needed to address and reduce the growing health and economic burdens caused by T2D.

While both genetic and various lifestyle factors have been identified as significant contributors to T2D development [5, 6], only some risk factors can be modified, making their investigation particularly important for T2D prevention. Several large, randomized trials have performed lifestyle interventions to prevent T2D [7–10], but they often focused on individuals with glucose impairments and involved few participants with short follow-ups. Therefore, large prospective studies are of interest to understand the long-term impact of lifestyle factors on T2D [11]. While numerous observational studies have examined the relation of single factors such as diet [12, 13], physical activity [14, 15], avoidance of cigarette smoking [16, 17], and moderate alcohol consumption [18, 19] to T2D, an increasing number of studies have also investigated the impact of combined lifestyle factors on T2D [20–23]. Utilizing a composite lifestyle risk index provides a more comprehensive assessment of overall lifestyle by capturing the cumulative effect of multiple behaviors, which often interact and coexist, such as unhealthy diet and low physical activity [24]. This approach is particularly advantageous as it can identify individuals at higher risk more effectively and facilitates the design of holistic intervention strategies targeting multiple behaviors simultaneously.

A systematic review and meta-analysis of cohort studies [20] showed that individuals adhering to the maximum combination of lifestyle-related low-risk factors (maintaining a healthy weight, healthy diet, regular exercise, not smoking, and low alcohol consumption) had an 80% reduced risk of T2D compared with the minimum reported (zero to three of these risk factors). In addition to individuals of European ancestry, the review also covered studies in Chinese populations and one study with Native Hawaiians and Japanese Americans [20] and highlighted the need to consider a greater range of diverse populations. Our study broadens this scope by examining a highly mixed population of individuals with European American, African American, Native Hawaiian, Japanese American, and Latino background. Identifying populations with unhealthy lifestyles could help design tailored public health interventions. Evidence from the Multiethnic Cohort (MEC) Study revealed important disparities in the prevalence of lifestyle risk factors for T2D across ethnic groups [25]. Additionally, the cross-sectional Adiposity Phenotype Study (APS), a subset of the MEC, found ethnic-specific differences in the cross-sectional association between a Lifestyle Risk Factor Index (LSRI) with T2D prevalence [21]. To explore these findings prospectively, the current analysis examined a healthy lifestyle as defined by LSRI on T2D risk across different ethnic groups in the large MEC Study.

## Methods

### Study population

The MEC is a prospective population-based cohort of 215,903 individuals aged 45–75 years, including Japanese American, European American, Latino, African American, and Native Hawaiian participants. The cohort was established in Hawaii and California in 1993–1996 to investigate diet, lifestyle and genetic risk factors for cancer and other chronic diseases by mailing the baseline questionnaire to individuals identified from drivers’ licenses and voter registration files as well as Health Care Financing Administration files [26]. Those who returned the self-administered questionnaire entered the cohort. In California, a Spanish version was available to improve accessibility and engagement among Latino participants of whom many were first generation immigrants. Cohort members provided information on demographics, anthropometrics, smoking status, medical history, physical activity, and diet [26]. Ethnicity was assessed using the question: “What is your ethnic or racial background?” with participants instructed to mark all that apply from the following options: Black or African American, Chinese, Filipino, Hawaiian, Japanese (includes Okinawan), Korean, Mexican or other Hispanic, White or Caucasian, and allowing them to write in any other background. Individuals reporting mixed ancestry, i.e., several backgrounds, were assigned to 1 of 6 groups according to the following priority ranking: African American, Native Hawaiian, Latino, Japanese American, European American, and Other. Body mass index (BMI, in kg/m^2^) was computed from self-reported weight and height. Follow-up questionnaires periodically updated information from surviving cohort members [27] who are followed until death, ascertained via states’ vital statistics and the National Death Index.

### Ascertainment of T2D status

T2D status was identified using self-reports by questionnaire and Medicare claims data. Self-reported diagnoses were assessed at cohort entry (Q1) and from four subsequent follow-up questionnaires (Q2-Q5), where MEC members were asked if they had ever been diagnosed with diabetes by their doctor. Participants reporting a T2D diagnosis and/or the use of diabetes medication (oral antidiabetics or insulin) in Q2-Q5 or in the biorepository questionnaire (2001–2006) were classified as T2D cases. Medicare claims for 1999–2016 were obtained for cohort members aged 65 and above with fee-for-service plans (58%) but not for those with managed care plans [25]. Medicare diagnoses were computed based on a Chronic Condition Warehouse (CCW) algorithm [28, 29] found to have high predictive values [30, 31]. The onset date of T2D was taken as the first report by questionnaire or Medicare. Any reports after Q1 and before December 31, 2016 were considered incident T2D diagnoses.

### Lifestyle risk factor index (LSRI)

As previously described for a subset of the MEC [21], all LSRI components, i.e., regular diet, alcohol consumption, smoking and physical activity were assessed by questionnaire (Q1) at cohort entry. Diet was determined using a semiquantitative validated food frequency questionnaire (QFFQ) [26]. The QFFQ included foods that accounted for over 85% of the nutrient intake of interest for each ethnic group [32]. Alcohol intake was categorized as number of drinks per day. Smoking was categorized into “current”, “former” or “never” smoking at cohort entry. Summary variables for moderate to vigorous physical activity were computed based on sedentary, moderate, and vigorous activities typically undertaken in a day [26].

The LSRI, initially developed in a UK Biobank study to assess the relation between lifestyle and dementia [33], is a composite score ranging from 0 to 4. After taking into account population-based differences in the current study, one point was given for not smoking currently, engaging in moderate to vigorous physical activity for a minimum of 150 min weekly, consumption of fewer than 1 (women) or 2 (men) alcoholic beverages per day, and adherence to at least 3 out of 7 food group recommendations. The dietary recommendations, aligned with cardiometabolic health recommendations [34], included adherence to ≥ 3 servings per day of fruits, ≥ 3 servings per day of vegetables, ≥ 3 servings per day of whole grains, ≥ 2 servings per week of fish, ≤ 1.5 servings per day of refined grains, ≤ 1 serving per week of processed meat, and ≤ 1.5 serving per week of non-processed red meat. One serving was defined as one cup of fruits or vegetables or one ounce (28.35 g) of grains, fish, or meat [34].

### Statistical analysis

**Fig. 1:**
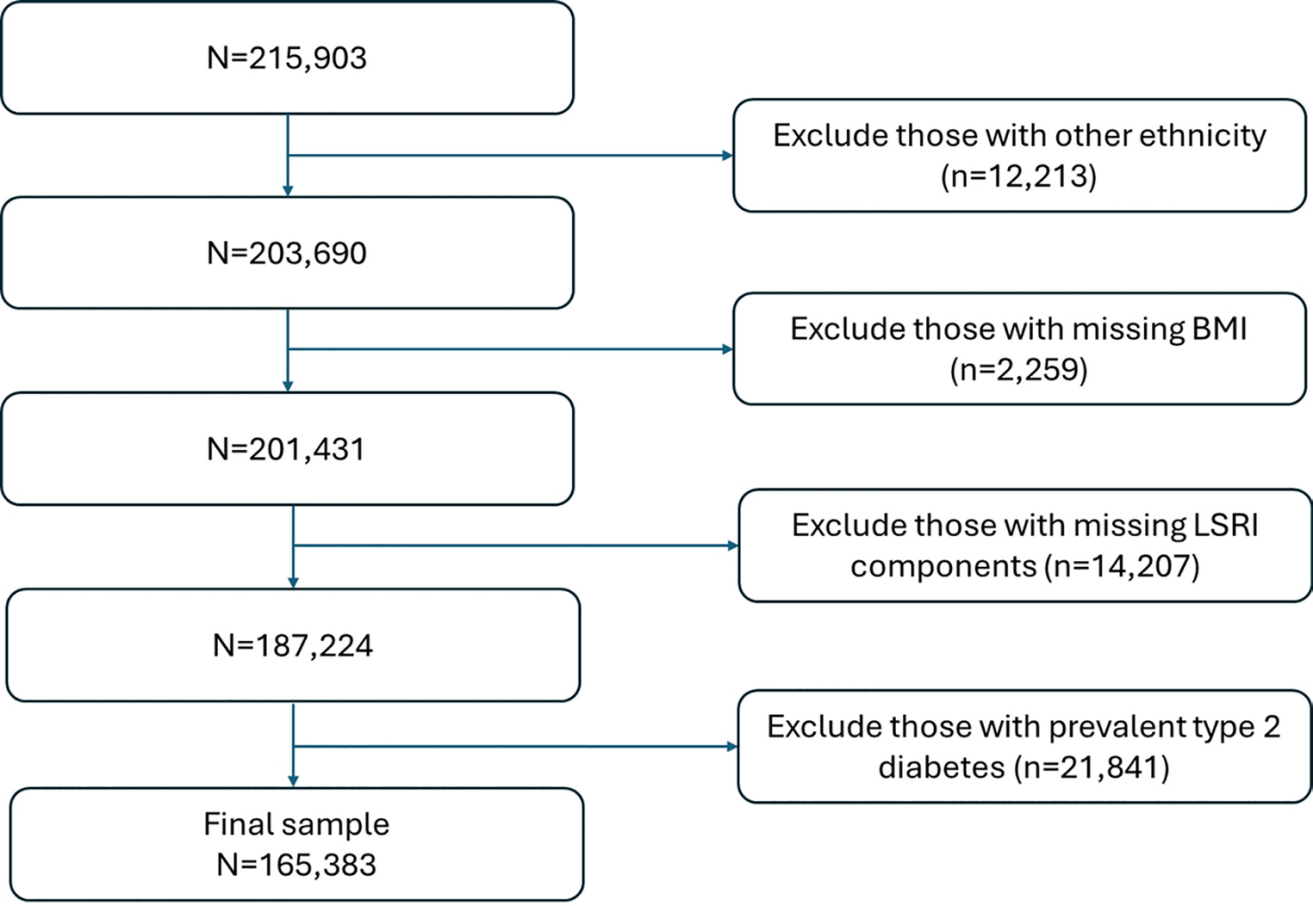
Study population flow chart of sample exclusions

**Fig. 2:**
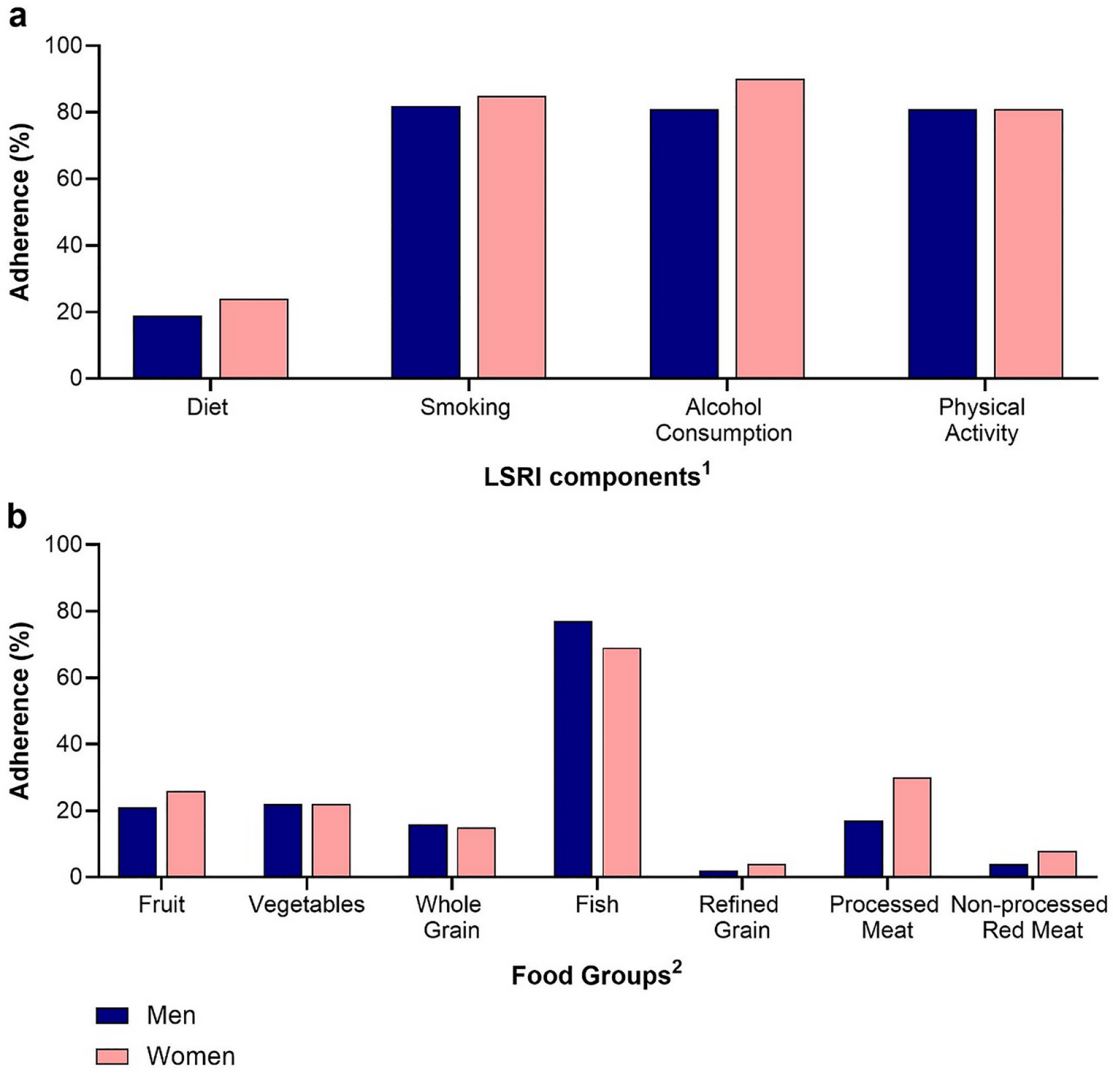
Adherence to LSRI components and dietary recommendations (%) by sex. Adherence by sex: (**a**) LSRI components and (**b**) dietary recommendations in percent (%) ^1^≥3 of 7 foods, no current smoking, ≤ 2 (men) or ≤ 1 (women) alcoholic drinks/day, ≥ 150 min/week physical activity ^2^Servings: ≥3 per day of fruits, ≥ 3 per day of vegetables, ≥ 3 per day of whole grains, ≥ 2 per week of fish, ≤ 1.5 per day of refined grains, ≤ 1 per week of processed meat, and ≤ 1.5 per week of non-processed red meat

**Fig. 3:**
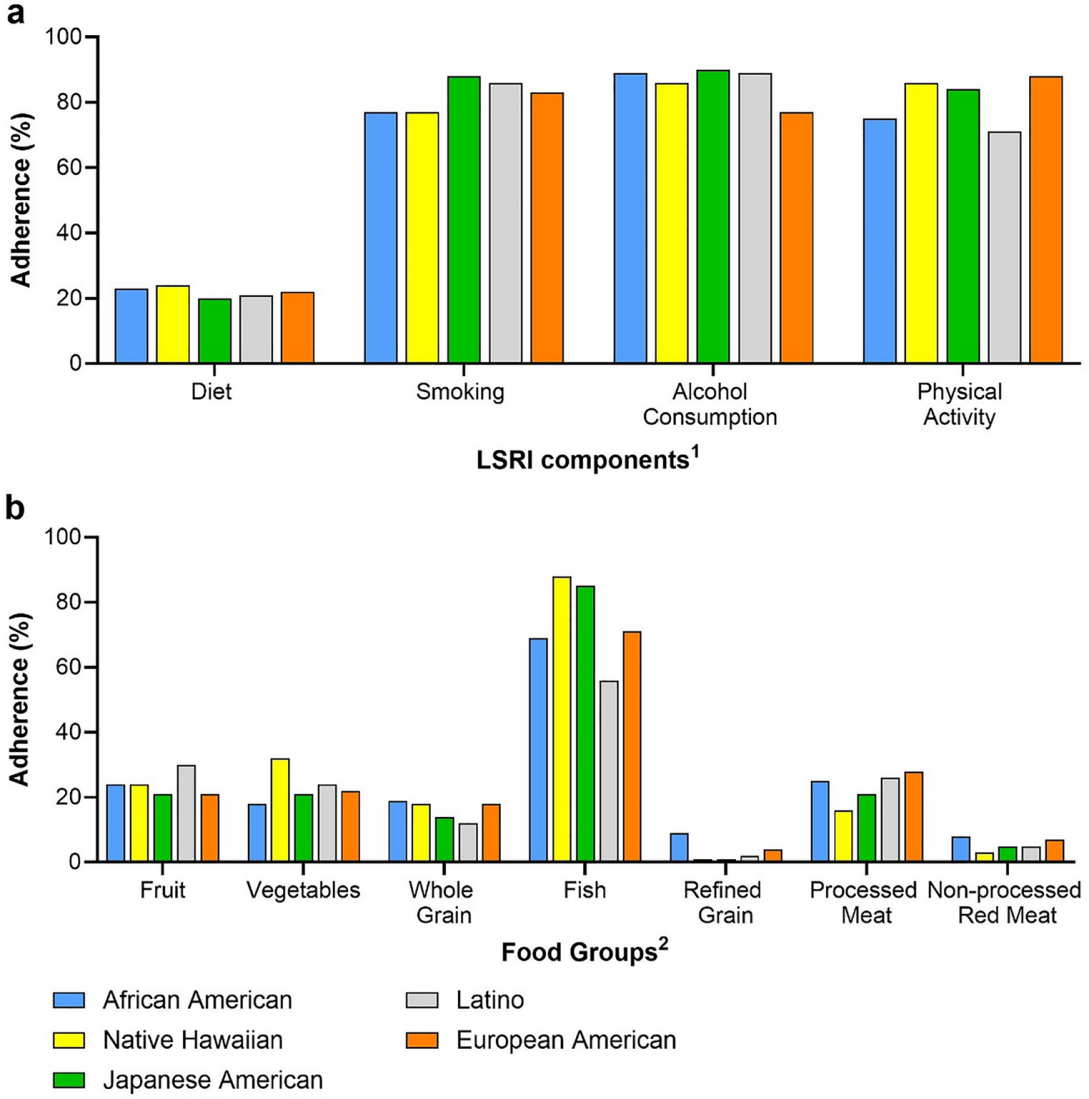
Adherence to LSRI components and dietary recommendations (%) by ethnic group. Adherence by ethnic group: (**a**) LSRI components and (**b**) dietary recommendations in percent (%) ^1^≥3 of 7 foods, no current smoking, ≤ 2 (men) or ≤ 1 (women) alcoholic drinks/day, ≥ 150 min/week physical activity ^2^Servings: ≥3 per day of fruits, ≥ 3 per day of vegetables, ≥ 3 per day of whole grains, ≥ 2 per week of fish, ≤ 1.5 per day of refined grains, ≤ 1 per week of processed meat, and ≤ 1.5 per week of non-processed red meat

All data management and analyses were conducted using SAS version 9.4 (SAS Institute, Cary, North Carolina, United States). Figure 1 was created using Microsoft PowerPoint, Microsoft 365 version 2504. Figures 2 and 3 were created using GraphPad Prism version 10.3.1 (GraphPad Software, Boston, Massachusetts, United States). Out of the 215,903 cohort members, first those with ethnic backgrounds other than African American, European Americans, Native Hawaiian, Japanese American, and Latino (*n* = 12,213) were excluded, then those with missing BMI (*n* = 2,259), then everyone with missing information to compute the LSRI (*n* = 14,207) and finally participants with prevalent T2D at cohort entry (*n* = 21,841) were excluded (Fig. 1). The final dataset consisted of 165,383 participants. Missing values for educational status (*n* = 688) were coded as a separate category to prevent further reduction of the analytic dataset.

Cox proportional hazards regression with sex and ethnic group as strata variables was used to evaluate the association of LSRI index with T2D incidence. The underlying time metric was the number of years from cohort entry until T2D diagnosis, death, or the end of the study (December 31, 2016). This approach generated hazard ratios (HRs) and 95% confidence intervals (95% CIs). Model 1 included baseline age (continuous) and education (< 12, 12–15, 16 + years) and Model 2 also BMI (18.5-<25, 25-<30, > 30 kg/m^2^) as covariates. The analyses of individual LSRI factors were stratified by sex and ethnic group and adjusted for the other LSRI factors in addition to model 1 or 2. Additionally, ethnicity-specific and sex-specific analyses were performed. We conducted a sensitivity analysis by recalculating the LSRI score based on the factors that showed an inverse association with type 2 diabetes in both models during individual component analyses. This was done to see which factors primarily drive the LSRI.

## Results

Among the total study population of 74,873 men and 90,510 women (Table 1), 44,518 (27%) were newly diagnosed with T2D during a mean follow-up of 17.2 ± 6.9 years. The largest ethnic group was Japanese Americans (29%), followed by European Americans (27%), Latino (22%), African Americans (16%) and Native Hawaiians (7%). The median and mean LSRI scores were 3 and 2.73 ± 0.79 points, respectively, with the following distribution: 7% 0/1 point, 28% 2, 51% 3, and 14% 4 points. By ethnic groups, the median LSRI score was 3 points for all, whereas the means differed to some degree with 2.82 points for Japanese Americans, 2.74 points for Native Hawaiians, 2.72 points for European Americans, 2.67 points for Latinos, and 2.66 points for African Americans.

**Table 1:** Characteristics of the study population by LSRI category

Characteristic	Category	ALL		LSRI 0/1		LSRI 2		LSRI 3		LSRI 4	
		*N*	%	*N*	%	*N*	%	*N*	%	*N*	%
Number		165,383	100	11,424	7	45,592	28	84,488	51	23,879	14
Incident T2D	No	120,865	73	8,554	7	33,063	27	61,509	51	17,739	15
	Yes	44,518	27	2870	6	12,529	28	22,979	52	6,140	14
Area	Hawaii	82,020	50	5,234	6	21,091	26	42,702	52	12,993	16
	Los Angeles	83,363	50	6,190	7	24,501	29	41,786	50	10,886	13
Sex	Men	74,873	45	7,014	9	22,634	30	36,335	49	8,890	12
	Women	90,510	55	4,410	5	22,958	25	48,153	53	14,989	17
Ethnicity	African American	26,124	16	2,493	9	7,713	30	12,182	47	3,736	14
	Native Hawaiian	11,476	7	890	8	3,104	27	5,623	49	1,859	16
	Japanese American	47,961	29	2,268	5	11,277	24	27,084	57	7,332	15
	Latino	35,889	22	2,454	7	11,280	31	17,770	50	4,385	12
	European American	43,933	27	3,319	7	12,218	28	21,829	50	6,567	15
BMI	< 18.5 kg/m^2^	3,069	2	308	10	847	28	1,396	45	518	17
	18.5-<25 kg/m^2^	69,154	42	5,030	7	17,726	26	35,073	51	11,325	16
	25-<30 kg/m^2^	63,456	38	4,247	7	18,193	29	32,631	51	8,385	13
	> 30 kg/m^2^	29,704	18	1,839	6	8,826	30	15,388	52	3,651	12
Education^1^	< 12 years	69,140	42	5,485	8	21,080	30	33,642	49	8,933	13
	12–15 years	49,567	30	3,602	7	13,362	27	25,429	51	7,174	15
	16 + years	45,988	28	1,964	4	9,792	21	26,208	57	8,024	17
Diet^2^	< 3 of 7 foods	129,804	78	11,315	9	44,083	34	74,406	57	0	0
	≥ 3 of 7 foods	35,579	22	109	0.3	1,509	4	10,082	28	23,879	67
Smoking	Current	26,890	16	9,551	35	14,948	56	2,391	9	0	0
	Not current	138,493	84	1,873	1	30,644	22	82,097	59	23,879	17
Alcohol consumption	> 1 or 2 drinks/day	22,985	14	7,726	34	12,265	53	2,994	13	0	0
	≤ 1 or 2 drinks/day	142,398	86	3,698	3	33,327	23	81,494	57	23,879	17
Physical activity	< 150 min/week	31,329	19	6,744	21	19,888	64	4,697	15	0	0
	≥ 150 min/week	134,054	81	4,680	3	25,704	19	79,791	60	25,285	18
		Mean	Std	Mean	Std	Mean	Std	Mean	Std	Mean	Std
Age at cohort entry (years)		59.5	8.9	58.3	8.6	59.0	8.8	59.6	8.9	61.0	8.8
Age at T2D diagnosis (years)		71.4	8.5	69.7	7.9	70.6	8.2	71.6	8.7	73.0	8.6
Follow-up time (years)		17.2	6.9	15.5	7.2	16.7	7.0	17.6	6.7	17.7	6.6
Total energy intake (kcal)		2,177	1,050	2,399	1,134	2,126	1,005	2,051	963	2,609	1,239

^1^Education 688 missing

^2^Adherence to ≥ 3 servings per day of fruits, ≥ 3 servings per day of vegetables, ≥ 3 servings per day of whole grains, ≥ 2 servings per week of fish, ≤ 1.5 servings per day of refined grains, ≤ 1 serving per week of processed meat, and ≤ 1.5 serving per week of non-processed red meat. One serving was defined as one cup of fruits or vegetables or one ounce (28.35 g) of grains, fish, or meat

Adherence was highest for moderate alcohol consumption (86%) and no smoking (84%), followed by physical activity (81%) but only 22% scored for diet adherence (Table 1). Adherence rates were higher for women than men: 24% vs. 19% for diet, 85% vs. 82% for smoking, and 90% vs. 81% for alcohol consumption but similar for physical activity (81%) (Fig. 2a). Diet adherence was low across ethnic groups (20-24%), while no smoking was lower in African Americans and Native Hawaiians (both 77%) compared to others (83–88%). All ethnic groups had higher adherence (86–90%) to the moderate alcohol intake than European Americans (77%). Physical activity adherence was lower among Latinos (71%), followed by African Americans (75%) compared to others (84–88%) (Fig. 3a). Adherence rates to most dietary recommendations were low in all participants, in particular, for refined grains (3%) and non-processed red meat (6%). Values for processed meat (24%), fruits (24%), vegetables (22%), whole grains (15%) were intermediate, whereas adherence was highest for fish consumption (73%). Sex differences of > 5% were observed (Fig. 2b) for adherence to fruit, fish, and processed meat recommendations, with higher adherence for women. Ethnic differences for dietary components (Fig. 3b) included a higher adherence to refined grain, processed and red meat recommendations among African Americans, a higher adherence to vegetable and fish recommendations among Native Hawaiians, low adherence to fruits and refined grains and high adherence to fish intake recommendations among Japanese Americans. Latinos had the highest fruit intake adherence while European Americans had the highest adherence for processed meats.

Comparing extreme LSRI categories (4 vs. 0/1 points) (Table 2), a 16% lower T2D risk (HR = 0.84; 95% CI 0.80–0.88) was observed in the fully adjusted Model 2. For each 1-point increase in LSRI score, the incidence of T2D was 6% lower (HR = 0.94; 95% CI 0.93–0.95) with a consistent association among men and women. For individual LSRI components, no current smoking and physical activity were associated with a significant 17% and 15% lower T2D incidence, whereas moderate alcohol intake predicted a 19% higher T2D risk. Models excluding BMI as a covariate (Model 1) yielded similar findings to the BMI-adjusted model except for diet showing a weak association not seen in Model 2. Given that smoking and physical activity were the only factors with significant inverse associations in both models, we recalculated the LSRI score based on these components for a sensitivity analysis. This recalculated score showed HRs of 0.84 (95% CI 0.82–0.86) in Model 1 and 0.83 (95% CI 0.81–0.84) in Model 2 comparing the higher to the lower category.

**Table 2:** T2D incidence in relation to the composite LSRI score and individual LSRI components

Group	Person-Years	T2D Cases	Category	Model 1^2^			Model 2^3^		
				HR^1^	95% CI		HR^1^	95% CI	
All	2,846,874	44,518	LSRI cont.^4^	0.94	0.93	0.96	0.94	0.93	0.95
	152,150	2,440	LSRI 0/1	1.00	--	--	1.00	--	--
	691,161	11,296	LSRI 2	1.00	0.96	1.04	0.93	0.89	0.97
	1,556,061	24,220	LSRI 3	0.93	0.90	0.97	0.86	0.83	0.90
	447,502	6,562	LSRI 4	0.86	0.82	0.90	0.84	0.80	0.88
Men	1,230,491	20,632	LSRI cont.^4^	0.96	0.94	0.98	0.96	0.94	0.97
	87,461	1,466	LSRI 0/1	1.00	--	--	1.00	--	--
	329,882	5,514	LSRI 2	0.97	0.92	1.02	0.92	0.87	0.97
	651,498	11,130	LSRI 3	0.95	0.91	1.00	0.89	0.85	0.94
	161,650	2,522	LSRI 4	0.86	0.81	0.91	0.85	0.80	0.90
Women	1,616,382	23,886	LSRI cont.^4^	0.93	0.91	0.94	0.93	0.91	0.95
	64,689	974	LSRI 0/1	1.00	--	--	1.00	--	--
	361,279	5,782	LSRI 2	1.01	0.95	1.08	0.94	0.88	1.00
	904,562	13,090	LSRI 3	0.90	0.85	0.96	0.83	0.78	0.88
	285,852	4,040	LSRI 4	0.85	0.80	0.91	0.83	0.77	0.88
All	2,846,874	44,518	Smoking^5^	0.90	0.87	0.92	0.83	0.81	0.85
	2,846,874	44,518	Alcohol consumption^5^	1.25	1.21	1.29	1.19	1.15	1.22
	2,846,874	44,518	Physical activity^5^	0.81	0.79	0.83	0.85	0.83	0.87
	2,846,874	44,518	Diet^5^	0.94	0.92	0.96	0.98	0.96	1.01

^1^Obtained by Cox regression with years between cohort entry and T2D diagnosis, death, or end of study as time metric, ethnicity and sex as strata variable for All and ethnicity only as strata variable for Men and Women

^2^Model 1: Adjusted for age and educational status

^3^Model 2: Same as Model 1 plus BMI as additional covariate

^4^LSRI as continuous variable

^5^In the individual analysis of LSRI components, all four components were included in the model

Across ethnic groups, 4 vs. 0/1 points were associated with a significantly lower T2D risk only among African American (27%), Latino (18%), and European American (14%) cohort members, but the strength of the associations was similar in the two models (Table 3). Looking at Model 2, the HRs for T2D for overweight (25-<30 kg/m^2^) were 1.80 (95% CI 1.75–1.84) and 3.11 (95% CI 3.03–3.19) for obesity (>30 kg/m^2^). The respective values were highest in European Americans (HR = 2.06; 95% CI 1.95–2.17 and HR = 4.22; 95% CI 3.99–4.46) followed by Native Hawaiians (HR = 2.01; 95% CI 1.81–2.22 and HR = 3.68; 95% CI 3.33–4.06), and Japanese Americans (HR = 1.83; 95% CI 1.77–1.90 and HR = 3.29; 95% CI 3.11–3.48). Latinos showed lower HRs of 1.55 (95% CI 1.48–1.63) and 2.56 (95% CI 2.42–2.69), whereas African Americans had the lowest HRs of 1.52 (95% CI 1.42–1.62) and 2.29 (95% CI 2.14–2.44).

**Table 3:** Association of T2D incidence with the LSRI score by ethnicity

Group	Person-Years	T2D Cases	Category	Model 1^2^			Model 2^3^		
				HR^1^	95% CI		HR^1^	95% CI	
African American	419,864	7,397	LSRI cont.^4^	0.91	0.89	0.94	0.90	0.87	0.92
	30,750	594	LSRI 0/1	1.00	--	--	1.00	--	--
	107,100	1,985	LSRI 2	0.94	0.86	1.02	0.88	0.81	0.96
	213,530	3,709	LSRI 3	0.84	0.77	0.92	0.78	0.71	0.84
	68,484	1,109	LSRI 4	0.77	0.70	0.85	0.73	0.67	0.81
Native Hawaiian	189,566	3,581	LSRI cont.^4^	1.03	0.99	1.07	0.99	0.95	1.03
	11,715	210	LSRI 0/1	1.00	--	--	1.00	--	--
	46,320	834	LSRI 2	1.02	0.89	1.18	0.93	0.81	1.07
	98,732	1,878	LSRI 3	1.06	0.93	1.21	0.91	0.79	1.04
	32,799	659	LSRI 4	1.09	0.94	1.27	0.95	0.81	1.10
Japanese American	833,321	13,615	LSRI cont.^4^	0.97	0.95	0.99	0.98	0.96	1.00
	30,571	482	LSRI 0/1	1.00	--	--	1.00	--	--
	171,010	2,877	LSRI 2	1.06	0.97	1.16	1.00	0.91	1.09
	498,075	8,157	LSRI 3	1.03	0.95	1.12	0.97	0.89	1.06
	133,665	2,099	LSRI 4	0.96	0.87	1.05	0.95	0.86	1.04
Latino	615,851	11,226	LSRI cont.^4^	0.92	0.90	0.94	0.92	0.90	0.95
	32,573	650	LSRI 0/1	1.00	--	--	1.00	--	--
	164,894	3,299	LSRI 2	0.99	0.92	1.07	0.96	0.88	1.03
	332,952	5,832	LSRI 3	0.87	0.81	0.94	0.85	0.79	0.92
	85,432	1,445	LSRI 4	0.83	0.75	0.90	0.82	0.75	0.90
European American	788,272	8,699	LSRI cont.^4^	0.95	0.92	0.97	0.95	0.93	0.98
	46,542	504	LSRI 0/1	1.00	--	--	1.00	--	--
	201,836	2,301	LSRI 2	1.00	0.91	1.09	0.91	0.83	1.00
	412,771	4,644	LSRI 3	0.96	0.88	1.05	0.87	0.79	0.95
	127,123	1,250	LSRI 4	0.85	0.77	0.94	0.86	0.78	0.95

^1^Obtained by Cox regression with years between cohort entry and T2D diagnosis, death, or end of study as time metric, sex as strata variable

^2^Model 1: Adjusted for age and educational status

^3^Model 2: Same as Model 1 plus BMI as additional covariate

^4^LSRI as continuous variable

## Discussion

This longitudinal analysis revealed an inverse relation between a composite LSRI and T2D risk with a 6% lower incidence of T2D per 1-point increase in LSRI score, but this association was only observed among African American, Latino, and European American participants. Before BMI adjustment, adherence to a healthy diet, physical activity, and no current smoking were inversely associated with T2D risk but adding BMI as covariate eliminated the association for diet. Notably, a score including physical activity and smoking alone showed slightly stronger associations with T2D risk compared to the score including all four components, indicating that these factors primarily drive the LSRI. The fact that the risk estimates for overweight and obesity were higher than for the LSRI confirms the important role of excess body weight in T2D development. Adherence to dietary recommendations was very low at 22%, compared to the other lifestyle factors (81–86%), with values below 50% for all dietary components except fish consumption. This may explain the surprisingly low inverse association between dietary adherence and T2D risk. The low adherence across most dietary components suggests that its potential benefits may not be fully realized without higher compliance although it is a critical factor. The high adherence to moderate alcohol consumption in all ethnic groups except European Americans suggests cultural influences.

Population surveys from European countries also reported similarly low adherence (3.8–38.5%) to a healthy diet as observed in our study, whereas engaging in adequate physical activity varied (17.6–76%) and were generally reported lower than in our study [35–39]. Similar to our findings, less than a quarter consumed alcohol above the recommended levels [37, 38] and *≥* 80% did not currently smoke in European cohort studies [37, 39, 40]. As also reported for a subset of the MEC [21], fish intake had the highest compliance at 73% in the current study, likely influenced by the proximity of Hawaii and California to the ocean.

The moderate reduction in T2D risk in our analysis is consistent with findings from other studies highlighting the beneficial impact of diet [12, 13], physical activity [14, 15] and avoidance of cigarette smoking [16, 17] on T2D development. However, in the current analysis, a positive association between moderate alcohol consumption and T2D risk was detected, which contrasts with previous reports [18, 19]. It should be noted, however, that the relation between alcohol consumption and T2D risk may be nonlinear and may vary by sex, ethnicity and the definition of abstainers (never drinking or noncurrent drinking). Furthermore, comparisons are often made between abstainers and light or moderate consumers [18, 19]. In this analysis, the “moderate consumers” group also included abstainers and was compared to alcohol consumers with relatively low intake compared to other populations.

Comparing our findings with previous studies on combined lifestyle behaviors and T2D is challenging due to variations in the combinations, assessment methods, and definitions of lifestyle factors. In a recent meta-analysis examining the relationship between combined lifestyle risk factors and T2D, which included 22 prospective cohorts with a total of 1,693,753 participants and 75,669 new cases of T2D, the definition of the “healthy diet” component varied significantly across studies. It ranged from daily vegetable consumption only to taking into account healthy dietary pattern scores including a variety of foods, such as vegetables, fruits, nuts and legumes, sugar-sweetened beverages, fruit juices, sodium, certain fats, whole grains and red/processed meat [20]. Many cohort studies used a composite score that weighted obesity equally with behaviors such as smoking, physical activity, and diet [20, 23]. However, it should be noted that obesity is recognized as a disease rather than a behavior [41]. The LSRI’s significant association with T2D risk persisted even after adjusting for BMI, indicating its independent significance. However, the influence of overweight and obesity on T2D, when adjusted for LSRI, was notably stronger. This underscores that while LSRI captures key lifestyle factors affecting T2D risk, BMI remains the strongest predictor. An example of a study investigating the effect of lifestyle factors independent from obesity (as well as genetic predisposition) on risk of T2D is a case-cohort study nested within the Diet, Cancer, and Health cohort [22]. Regardless of genetic risk, both obesity and an unfavorable lifestyle were independently associated with an increased risk of developing T2D [22]. Notably, an unfavorable lifestyle showed a modest association with an 18% increase in T2D risk, a finding in accordance with the 16% reduction in T2D risk for a beneficial lifestyle in the current study. The substantial 85% reduction in T2D risk reported by the meta-analysis included obesity as one of the five risk factors [20].

Many of the previous studies on combined lifestyle risk factors and T2D were conducted in individuals of European origin [20]. The current findings suggest, however, noteworthy differences across ethnic groups. The LSRI was significantly inversely associated with T2D risk in participants of European American, African Americans, and Latino ancestry. However, no significant associations were observed in Japanese Americans and Native Hawaiians, which is consistent with findings reported by the cross-sectional APS that examined T2D prevalence independent of body fat distribution [21]. Subgroup analyses in a meta-analysis also showed significant differences in relative risk for extreme lifestyle risk factors comparisons and T2D incidence based on race/ethnicity, although the meta-regression did not reveal any significant differences between any two races/ethnicities [20]. In a previous prospective MEC analysis, several risk factors showed significant ethnic-specific associations with T2D risk; for instance, physical activity was not related to T2D risk in Japanese Americans [25]. It should be noted that BMI has a substantial impact on T2D risk among Japanese Americans due to their Asian ancestry and among Native Hawaiians who often have high levels of Chinese admixture [42], which may make it challenging to identify additional risk factors. In models without BMI, the association between LSRI and T2D was significant for Japanese Americans, suggesting that BMI plays a critical role in influencing the relation between lifestyle behaviors and T2D risk in this group. The positive, although non-significant, association observed for Native Hawaiians is challenging to interpret; an imprecise positive association of LSRI with T2D was, however, also reported by the cross-sectional APS [21]. As Native Hawaiians constitute the smallest group with only 7% of participants, the statistical power was low. Differences in T2D etiology by ethnic group, in particular in persons of Asian descent and Native Hawaiians, who have significant Asian admixture [43], may exhibit a unique pathogenesis for T2D as they are more prone to accumulating abdominal visceral fat [44], which increases their risk for insulin resistance compared to abdominal subcutaneous fat [45]. Additionally, a higher insulin sensitivity and lower insulin response in East Asians compared to individuals of European ancestry contribute to T2D risk [46].

The study’s strengths include its focus on a multiethnic population with four ethnic groups who experience substantially higher T2D incidence rates than individuals of European ancestry [42], a large sample size of newly diagnosed T2D cases and a more than 20 years follow-up. Additionally, the collection of lifestyle information before T2D diagnosis helps to avoid recall bias, enhancing the credibility of our findings. Since the LSRI generally assumes an additive effect without confirming whether each component has the same impact on T2D risk [20], we examined the influence of individual score components to understand their distinct influence, something that was rarely done in previous studies [20]. Including a composite score instead of individual lifestyle factors has the advantage of reflecting the combined and synergetic influences of these factors, and the specific score used here aggregates common and easily quantifiable behaviors, offering a practical and holistic assessment of T2D risk.

Limitations included the fact that the LSRI dietary recommendations applied in the current study align with general cardiometabolic health guidelines, but they were not specifically tailored for T2D, such as considering specific dietary factors like sugar-sweetened beverages [47]. Additionally, the LSRI assumes equal influence of the lifestyle components on disease development, potentially leading to weaker estimates for T2D [48]. Therefore, T2D-specific indices could offer more accurate evaluations [48]. Furthermore, the LSRI in this study focused on key lifestyle components but did not encompass all known risk factors for T2D, e.g., duration and quality of sleep [49, 50]. A further limitation of this study is the one-time assessment of risk factors without taking change in lifestyle behaviors over time into account. Self-reported lifestyle behaviors also may have been inaccurately reported. Although direct validation of T2D diagnoses through fasting glucose, HbA1c levels, or medical records was not possible, previous population-based studies have shown high validity in T2D self-reports, e.g., in the Women’s Health Initiative, 92% of self-reported prevalent and 82% of incident T2D cases were supported by medical records [30]. Similarly, over 80% of self-reported T2D cases were validated using Australian administrative data [51].

In summary, our study demonstrates significant ethnic differences in how lifestyle factors and BMI affect T2D incidence. An association for the LSRI was only detected in three out of five ethnic groups while the varying influence of BMI across ethnic groups underscores the importance of considering physiological differences, such as body fat distribution, in T2D development. Future studies need to investigate additional behaviors associated with T2D, account for lifestyle changes over time, and identify culturally tailored interventions to improve lifestyle behaviors in diverse populations as described elsewhere [52, 53].

## Data Availability

The data used in this study cannot be publicly shared due to privacy considerations as they contain identifiable patient information. However, researchers who meet the access criteria can request the data from the Multiethnic Cohort study (http://www.uhcancercenter.org/research/the-multiethnic-cohortstudy-mec/data-sharing-mec).
